# Active travel to non-school destinations but not to school is associated with higher physical activity levels in an ethnically diverse sample of inner-city schoolchildren

**DOI:** 10.1186/s12889-016-3920-1

**Published:** 2017-01-05

**Authors:** Lee Smith, Daniel Aggio, Mark Hamer

**Affiliations:** 1 The Cambridge Centre for Sport and Exercise Sciences, Department of Life Sciences, Anglia Ruskin University, Cambridge, CB1 1PT UK; 2 Physical Activity Research Group, University College London, London, UK; 3 National Centre for Sport and Exercise Medicine, Loughborough University, Loughborough, UK; 4 National Institute for Health Research Leicester-Loughborough Diet, Lifestyle and Physical Activity Biomedical Research Unit, Leicester, UK

**Keywords:** Active transport, Accelerometry, Sedentary behaviour

## Abstract

**Background:**

This study investigated the association of travel mode to school and non-school destinations with objectively assessed health markers and physical activity in an ethnically diverse sample of inner-city UK schoolchildren.

**Methods:**

We used data from the Camden Active Spaces project (*n* = 450 children aged 9.1 yrs) to examine associations of school travel mode and frequency of active travel to non-school destinations with daily and out-of-school physical activity, sedentary time and health markers; whilst controlling for appropriate covariates including objectively measured route length.

**Results:**

High frequency of active travel to non-school destinations was associated with more time in moderate-to-vigorous physical activity during out-of-school periods (3.8, 0.8–6.9 min/d) and greater out-of-school (738, 197.4–1278.6 steps/d) and daily step counts (588.1, 51.6–1124.6 steps/d). No associations were observed between school travel mode, health outcomes and activity levels.

**Conclusion:**

High frequency of active travel to non-school destinations is associated with higher levels of physical activity. These findings highlight the contribution of travel to non-school destinations to overall physical activity levels in schoolchildren.

**Electronic supplementary material:**

The online version of this article (doi:10.1186/s12889-016-3920-1) contains supplementary material, which is available to authorized users.

## Background

Physical activity has many benefits in young people, including promoting favourable levels of risk factors such as adiposity, blood pressure and triglyceride levels, [1–3] and maintenance of psychological health [4]. However, data from the Health Survey for England (2008) showed that just 24% of girls and 32% of boys aged 2 to 15 years achieved at least 60 min of moderate to vigorous intensity physical activity (MVPA) each day, the UK recommended physical activity guideline to maintain good health [5]. In addition to the current low levels of physical activity, specific physical activity behaviours in young people such as walking and cycling to school have declined in recent decades [6].

Using active modes of travel (ie, walking and cycling) may benefit the health of young people. In a systematic review, 55% of studies demonstrated an inverse association between active travel to school and weight status/body composition. In addition, the review identified 5 papers that suggested active travel may also be beneficial for cardio-respiratory fitness [7]. For example, Cooper et al. [8] found that children and adolescents who cycled to school were nearly five times more likely to be in the top quartile of fitness than those who used motorised modes. It may be that these differences in health between travel mode groups are a result of additional physical activity accumulated from using active modes of travel. However, the majority of studies identified in the review were of a cross-sectional design. These findings may therefore also be attributable to “healthy” young people choosing to use active modes of travel to school. Other components of fitness such as muscular strength and power have been shown to be positively associated with health during childhood [9–11]. Moreover, flexibility in childhood may also reduce future risk of chronic musculoskeletal problems [12]. It is plausible to assume that higher levels of active travel (a component of physical activity) during childhood may be favourably associated with these components but little research exists in this area.

Two systematic reviews have examined associations between mode of travel to school and overall physical activity in young people demonstrating that active modes of travel can equate to an additional 5 to 37 min/day in MVPA in comparison to those who use motorised transport (passive travel) [13, 14]. Nevertheless, some studies found null associations between travel mode and overall levels of physical activity [15, 16]. This may be explained by the young age (5 to 11 years) of the participants in the two studies. Younger children (but not older children) may only be allowed to travel to school by active modes when the school is located close to their home (therefore only a short distance may be travelled actively), or if they are accompanied by an adult (thus restricting spontaneous play en-route), therefore limiting younger children’s time spent in physical activity when using active modes of travel to school. More recently, Smith et al. found a change from a passive to an active mode of travel resulted in an increase in daily MVPA (mean increase: boys 9mins and girls 6mins) [17, 18]. Despite growing evidence on the association between mode of travel to school and physical activity levels, the association between mode of travel to non-school destinations (e.g. to a friend’s house or to the local shops) and physical activity levels is unknown. Thus, there is a need to broaden the research area into examining the role of active travel to non-school destinations. These journeys may provide an opportunity for young people to incorporate additional physical activity into their daily lives. For example, a study of 5 to 6 year old Australian children found that boys and girls walked or cycled for an average of seven trips per week, but only two trips, for both sexes, related to the school journey [19]. Furthermore, in the UK, non-school journeys made up more than 70% of all journeys made by young people under 17 years in 2008 [20]. To our knowledge only one study has examined the role of travel mode to non-school destinations in young people, showing that active travel to non-school destinations was associated with higher overall levels of physical activity, independent of travel mode to school [17]. However, this study used a rural sample that was not ethnically diverse (white ethnicity = 91%). The present study aims to investigate the association of travel mode (to non-school and school destinations) with health markers and physical activity levels in an ethnically diverse sample of inner-city primary and high school children residing in Camden, London, UK.

## Methods

Cross-sectional data from the Camden Active Spaces project [21]. In brief, participants (*n* = 450), were aged 5 to 15 years from 7 primary and 2 secondary schools, from the London borough of Camden, UK. Data were collected between June and November 2014 (during school term time). Parents of primary school children consented for them to participate via an opt-out approach. Participants in secondary schools also provided explicit written assent. Ethical approval was granted by the University College London Research Ethics Committee (Reference number: 4400/002).

### Exposure variables: frequency of active travel to non-school destinations and usual travel mode to school

Only one other study has investigated travel mode to non-school destinations [17] and therefore participants were asked similar questions to those asked in this study, “when you travel to the following places, how often do you walk or cycle to them? (please tick one box on each line).” Destinations were (a) friends’ houses, (b) parks, (c) shops and (d) sport facilities. Response options were: (a) never or rarely, (b) sometimes, (c) most of the time, (d) all of the time, and (e) it is not within walking distance. Participants completed items with the assistance of a research assistant. Items were explained to participants and participants were told to tick only one box for each item. An average frequency score for active travel to non-school destinations was calculated for each student, by assigning a numeric value to each response option ─ it is not within walking distance = 0, never or rarely = 1, sometimes = 2, most of the time = 3 and all of the time = 4 ─ and calculating a student’s mean score. Participants with a mean value ≤2.5 were assigned to the group “low frequency of active travel to non-school destinations” and those who reported >2.5 were assigned to the group “high frequency of active travel to non-school destinations.”

In the present study participants were also asked “how do you usually travel to school?” and “how do you usually get home from school?”. Travel options were: (a) bus, (b) car, van or taxi, (c) walking, (d) cycling and (e) skateboard, scooter or rollerblades. Walking, cycling and skateboard, scooter or rollerblades were grouped as active and other responses were grouped as passive. Participants completed items with the assistance of a research assistant. Items were explained to participants and participants were told to tick only one box for each item. Participants taking active modes of travel on one or both journeys to and from school were grouped as ‘active’ and those making both passively as ‘passive’.

### Outcome variables: physical activity and sedentary time

Participants were asked to wear tri-axial accelerometers (Actigraph GT3X), around the waist, for seven consecutive days, providing an objective measure of physical activity and sedentary time. Tri-axial accelerometers are considered valid and reliable for measuring these behaviours in young people [22]. The first day of accelerometery data, a partial day of wear time, was removed. To categorise time spent sedentary and in MVPA age-specific cut points were used [23]. Sedentary time was classified as less than 100 accelerometer counts per minute (cpm) and MVPA as more than 3000 cpm [23]. Greater than or equal to sixty minute bouts of continuous zero counts were considered as non-wear time and excluded from the data [24]. A daily average for time sedentary, in MVPA and number of steps taken were derived from valid days. A valid day required at least 500 min wear time between 07:00 and 00:00. Participants with one valid day or more were included in the analysis. This criteria has been used in similar studies [23]. Daily averages were also calculated for out-of-school time (including wear time during weekdays between 07:00 and 09:00 and 15:00 and 00:00, and on weekend days between 07:00 and 00:00). Participants required at least 140 min of out-of-school wear time during weekdays to be included in this analysis.

### Health and fitness outcomes

Body Mass and adiposity (%) were measured using a Tanita SC-330 Body Composition Analyser (Tanita Inc, Illinois, USA), by a trained research assistant. Grip strength was determined using the hand-held Dynamometer (JAMAR®, Hydraulic Hand Dynamometer), which was held in the dominant hand while participants were standing. Participants were then asked to squeeze the grip as hard as possible. Participants also performed a horizontal jump test, assessing leg power. From a standing position participants were instructed to bend their knees and engage their arms to jump forward and as far as possible. Flexibility was measured using a standard sit-and-reach box. Participants performed the sit-and-reach test with their shoes removed and their knees fully extended. Participants were asked to lean forward and as far as possible, while sliding the ruler along the surface of the box with their fingertips. A peak flow meter was used to assess peak expiratory flow. Each participant carried out each test three times and the highest score was recorded.

### Covariates

Participants’ age, sex, ethnicity, and school deprivation were recorded. School deprivation was derived from school postcodes. Geoconvert was used to translate postcodes into an Indices of Deprivation 2007 Lower Super Output Area (LSOA) Score [25]. Schools were then ranked in order of deprivation. Shortest network distance from home to school was calculated using a Geographic Information System (GIS). We also accounted for daylight hours according to the time of year that data was collected.

### Analyses

Associations of school travel (active vs. passive) and non-school travel (high frequency of active travel vs. low frequency), with daily and out-of-school physical activity (steps and time in MVPA) and sedentary time were examined using multiple linear regression models. Analyses were adjusted for pre-specified covariates that were hypothesised to be independently associated with exposure and outcome variables. These included age, Actigraph wear time, sex, body fat, ethnicity and area deprivation. Analyses using school travel mode as the main exposure also controlled for shortest network distance from home to school. Distance from home to school was positively skewed and thus log transformed prior to analyses. We also performed a sensitivity analysis including only participants with 3 or more valid days of accelerometry in the models (Additional file 1: Table S1). All analyses were conducted using SPSS version 20.

## Results

Of the initial 450 participants that took part, a total of 396 provided valid accelerometry data. Of these 396 participants, 55.8% provided at least five valid days, 36.4% provided 2–4 valid days and 7.8% provided only one valid day. Approximately 36% of the sample were White British. Participants without valid accelerometry data were significantly younger (8.4 yrs vs. 9.2 yrs, *p* < 0.05), were more likely to be male (62.1% vs. 48.0%), had lower levels of body fat (19.7% vs. 22.4%, *p* < 0.05) but did not differ by travel modes or ethnicity when compared to the remaining sample. Participants with missing travel and covariate data were also excluded leaving a final sample of 322 participants for analyses using non-school active travel as the main exposure variable. For analyses using school travel mode as the main exposure, the final sample reduced to 304 due to missing school travel data and missing or invalid postcodes. Median network distance from home to school was 904.7 (interquartile range, 488.6–1455.8) metres. Descriptive statistics for the study population can be found in Table 1, including health and fitness outcomes and objectively measured physical activity and sedentary behaviour. No significant differences between ethnicity (white versus non-white) and physical activity levels were observed (Additional file 1: Table S2). A total of 168/322 (52.2%) participants were classified as having a high frequency of active travel to non-school destinations. School active travel was reported in 233 children (76.6%) and 71 (23.4%) reported passive travel modes.

**Table 1:** Participant descriptive statistics by non-school active travel frequency (*n* = 322)

	Mean ± SD	
	Low (*n* = 154)	High (*n* = 168)
Age (years)	9.3 ± 2.3	9.7 ± 2.2
Body fat (%)	22.4 ± 7.9	22.8 ± 7.9
MVPA (mins/day)	27.6 ± 15.8	29.0 ± 18.4
Sedentary (mins/day)	368.8 ± 89.5	380 ± 89.8
Daily steps	9933.2 ± 2724.7	10208.1 ± 2753.1
Out-of-school MVPA (mins/day)^a^	14.5 (8.2, 24.5)	17.3 (8.9, 29.1)
Out-of-school sedentary (mins/day)	244.7 ± 91.5	244.0 ± 91.5
Out-of-school steps	6531.2 ± 2751.8	7150.7 ± 2741.5

^a^Out-of-school MVPA data presented as median and interquartile range

No associations between school travel mode, health markers and accelerometry data were observed (data not shown). Table 2 presents associations for active travel to non-school destinations. A high frequency of active travel to non-school destinations was associated with approximately 4 additional minutes per day of MVPA during out-of-school periods, which persisted after adjustment for all covariates (B 3.8, 95% CI 0.8 – 6.9 min/d). Active travel to non-school destinations was also associated with more than 700 additional steps during out-of-school periods (B 730.4, 95% CI 205.1, 1255.7) and more than 500 additional steps across the whole day (B 580.2 95% CI 60.4, 1100.0). However, there was no significant association between active travel to non-school destinations and total MVPA across the whole day. In an additional analysis, school travel mode was also included in models as a potential confounding variable; however, there were no substantial differences in results (data not shown). No associations between frequency of active travel non-school destinations and health markers were found (data not shown). Sensitivity analyses revealed no notable differences in the results when including only participants with 3 or more valid days of accelerometry (Additional file 1: Table S1).

**Table 2:** Association between frequency of active travel to non-school destinations (low vs. high frequency of active travel) and physical activity (*n* = 322)

	Model 1		Model 2	
	B coefficient (95% CI)	Beta	B coefficient (95% CI)	Beta
Out of school time (reference: low frequency of active travel)				
MVPA (mins/day)	3.2 (0.2, 6.3)*	0.11	3.8 (0.8, 6.8)*	0.13
Sedentary (mins/day)	−3.9 (−15.1, 9.2)	−0.02	−4.3 (−17.6, 9.1)	−0.02
Steps	776.1 (246.8, 1305.4)**	0.14	719.2 (201.8, 1236.7)**	0.13
Daily (reference: low frequency of active travel)				
MVPA (mins/day)	2.2 (−1.5, 5.9)	0.06	3.5 (−0.1, 7.0)	0.10
Sedentary (mins/day)	2.9 (−8.9, 14.7)	0.02	2.6 (−9.2, 14.5)	0.44
Steps	494.1 (−43.2, 1031.5)	0.09	576.2 (62.5, 1089.8)*	0.11

*Model 1* adjusted for age and device wear time. *Model 2* additionally adjusted for sex, school deprivation, ethnicity, daylight saving and body fat

**p < 0.01, *p < 0.05

## Discussion

The present study found in an ethnically diverse sample of inner-city London children that those who reported a high frequency of active travel to non-school destinations spent approximately four additional minutes a day during out-of-school time in MVPA than those who had a low frequency (B 3.8, 95% CI 0.8–6.9 min/d). Moreover, these children accumulated significantly greater overall and out-of-school step counts. This supports findings from the only previous study examining this association in a sample of predominantly white rural children; Smith et al. found that active travel to non-school destinations was associated with higher overall levels of physical activity with the greatest difference in MVPA being observed after school (between 15:00 and 20:00) [17]. This difference in MVPA may reflect the journeys themselves, spontaneous play during the journeys, or the encouragement of active behaviour in other areas of young people’s lives [14]. It is also possible that the difference in MVPA is owing to active children choosing to travel by active means. Further investigation into the mechanisms by which active travel may be associated with overall physical activity is needed.

The present study found no significant associations between travel mode to school and physical activity levels. This is in contrast with some previous literature, but not all [14]. Null findings have generally been found in younger age children (5 to 11 years), which was comparable to the age of children in the present study. It is also possible that the null association within the present sample may be owing to a “London effect,” that is young people in London may travel short distances to school with little opportunity for spontaneous play en-route and therefore the journey to school may have a small contribution to time spent in MVPA. However, we did control for objectively measured network distance from home to school.

In terms of health and fitness outcomes, the present study found no associations with school and non-school travel mode. This supports the argument that perhaps active travel has little impact on the health of inner-city schoolchildren due to the likely short distances from home to school and non-school destinations. These results contradict previous findings that show active travel is associated with health and fitness outcomes [7]; more research is required in this area using robust experimental design.

### Strengths and limitations

A clear strength of this study is its ethnically diverse sample of inner-city London children. A further strength is that we were able to control for network distance from home to school; however, we were unable to control for network distance from home to non-school destinations as this included multiple destinations that we did not have GIS data available for. Analyses in this study may be limited by the relatively crude measure used to assess travel mode to school and non-school destinations as they did not allow daily mode of travel to be ascertained. To our knowledge there is no published validation of questions to ascertain “usual travel mode” in young people. However, systematic reviews on travel mode and physical activity in young people identified that a large proportion of studies (see for example, [8, 26]) use usual travel mode questions to measure travel behaviour [13, 14]. Importantly, similar measures have also been used to investigate the association between mode of travel to non-school destinations and physical activity [17].

Accerlerometers underestimate the intensity of physical activity undertaken whilst cycling as they are calibrated to record ambulatory activity [27]. However, in our sample just 3.5% of children cycled to school. The present study was carried out in a borough of inner-city London such an area is likely to have a high level of walkability but also a high traffic volume. A high traffic volume may present a higher risk to children’s safety when traveling via an active mode. Subsequently, parents may limit the independence of their children to travel by active modes. However, traffic volume data was not available to consider in analytical models. Moreover, a high level of walkability may facilitate active travel but such data was not available. The structure of the travel to school and non-school items differed and thus may have produced different interpretations and potentially different responses if indeed the non-school item was asked for travel to school and vice versa. However, to allow the results of the present paper to be comparable to previous literature then one most ensure similar measures to previous literature are used. In the case of the present paper the non-school travel item mirrored that previously used as did the school travel item [17, 18].

## Conclusion

A high frequency of active travel to non-school destinations was associated with more time in MVPA during out-of-school periods. Significantly greater step counts over the whole day and during out-of-school time were also observed. Active travel to school was not associated with levels of physical activity or health and fitness outcomes. Our findings emphasise the importance of promoting active travel to non-school destinations to achieve higher physical activity levels in young people.

## Additional file

Additional file 1: Table S1. Association between frequency of active travel to non-school destinations (low vs. high frequency of active travel) and physical activity in participants with ≥ 3 days accelerometry (*n* = 273); **Table S2.**. Association between ethnicity and physical activity levels (white vs. non-white ethnicities). (DOCX 13 kb)

## Abbreviations

CPM: Counts per minute
LSOA: Lower super output area
MVPA: Moderate-to-vigorous intensity physical activity
SPEEDY: The sport physical activity and eating behaviour: environmental determinants in young people study
