# Supplementary material for: Active travel to non-school destinations but not to school is associated with higher physical activity levels in an ethnically diverse sample of inner-city schoolchildren
Source: BMC Public Health. 2017 Jan 5;17:13. doi: 10.1186/s12889-016-3920-1 (PMC5216598; doi:10.1186/s12889-016-3920-1)
Supplement: Additional file 1: Table S1. — Association between frequency of active travel to non-school destinations (low vs. high frequency of active travel) and physical activity in participants with ≥ 3 days accelerometry (n = 273); Table S2.. Association between ethnicity and physical activity levels (white vs. non-white ethnicities). (DOCX 13 kb) [file 12889_2016_3920_MOESM1_ESM.docx]

Additional file 1: Table S1. Association between frequency of active travel to non-school destinations (low vs. high frequency of active travel) and physical activity in participants with ≥ 3 days accelerometry (n = 273)

|  | **Model 1** | | **Model 2** | |
| --- | --- | --- | --- | --- |
|  | **B coefficient† (95% CI)** | **Beta** | **B coefficient† (95% CI)** | **Beta** |
| **Out of school time (reference:**  **low frequency of active travel)** | |  |  |  |
| MVPA (mins/day) | 3.3 (-0.1, 6.7) | 0.12 | 3.9 (0.6, 7.2)* | 0.14 |
| Sedentary (mins/day) | -9.4 (-22.7, 3.9) | -0.06 | -10.1 (-23.8, 3.6) | -0.06 |
| Steps | 813.4 (233.4, 1393.5)** | 0.15 | 718.2 (150.3, 1286.1)* | 0.14 |
| **Daily (reference:**  **low frequency of active travel)** | |  |  |  |
| MVPA (mins/day) | 2.8 (-1.2, 6.8) | 0.08 | 4.4 (0.5, 8.3) | 0.13 |
| Sedentary (mins/day) | -4.0 (-16.3, 8.4) | -0.02 | -3.8 (-16.5, 8.8) | -0.02 |
| Steps | 586.5 (9.5, 1163.5) | 0.11 | 683.1 (128.2, 1238.0)* | 0.13 |

**p<0.01, *p<0.05.

*^Model 1^* ^adjusted for age and device wear time.^ *^Model 2^* ^additionally adjusted for sex, school deprivation, ethnicity, daylight saving and body fat.^

|  | **B coefficient† (95% CI)** | **Beta** |
| --- | --- | --- |
| **Out of school time (reference:**  **White British)** | |  |
| MVPA (mins/day) | -1.4 (-4.9, 2.0) | -0.05 |
| Sedentary (mins/day) | -7.3 (-21.6, 7.0) | -0.04 |
| Steps | -490.8 (-1083.0, 101.4) | -0.09 |
| **Daily (reference:**  **White British)** | |  |
| MVPA (mins/day) | -0.4 (-4.4, 3.7) | -0.01 |
| Sedentary (mins/day) | 1.4 (-11.8, 14.5) | 0.01 |
| Steps | -137.1 (-715.8, 441.6) | -0.03 |

Additional file 1: Table S2. Association between ethnicity and physical activity levels (white vs. non-white ethnicities)

*^Model adjusted^* ^for sex, school deprivation, ethnicity, daylight saving and body fat.^
